# Betulinic Acid-Derived HIV-1 Maturation Inhibitors: Bridging Chemical Design, Molecular Dynamics, and Experimental Evidence

**DOI:** 10.3390/ijms27219509

**Published:** 2026-10-24

**Authors:** Ardak Sapiyeva, Nurlan Sadykov, Aziza Omari, Karlygash Akpayeva, Galiya Aipova, Kairat S. Zhakipbekov

**Affiliations:** 1 Department of General and Biological Chemistry, Astana Medical University, Astana 010000, Kazakhstan; sapieva.a@amu.kz; 2 School of Pharmacy, Astana Medical University, Astana 010000, Kazakhstan; omari.a@amu.kz; 3 Department of Pharmaceutical Disciplines, Astana Medical University, Astana 010000, Kazakhstan; akpayeva.k@amu.kz (K.A.); aipova.g@amu.kz (G.A.); 4 Department of Organization, Management and Economics of Pharmacy and Clinical Pharmacy, Asfendiyarov Kazakh National Medical University, Almaty 050012, Kazakhstan

**Keywords:** betulinic acid, bevirimat, HIV-1 maturation, CA–SP1, six-helix bundle, molecular dynamics, structure-activity relationships, structural biology, translational pharmacology

## Abstract

Betulinic acid-derived human immunodeficiency virus type 1 (HIV-1) maturation inhibitors connect natural-product medicinal chemistry with inhibition of capsid–spacer peptide 1 (CA–SP1) processing. This critical narrative review examines how chemical modifications relate to target structure, conformational dynamics, antiviral activity and development limitations. C-3 pharmacophore replacement and modification of the C-28/C-17 region improved antiviral breadth and exposure, but improved cellular potency alone did not establish clinical differentiation. Cryo-electron microscopy, microcrystal electron diffraction and nuclear magnetic resonance support engagement of the CA–SP1 six-helix bundle and restriction of its dynamics. Molecular simulations offer an ensemble-based explanation whose interpretation depends on assembly state, cofactors, sampling and experimental validation. Binding kinetics and cleavage measurements provide particularly informative connections between molecular observations and antiviral activity. Later-generation compounds demonstrate that broader susceptibility coverage and favorable pharmacokinetics must be evaluated alongside tolerability, resistance and regimen requirements. Progress therefore depends on aligning compound identity, relevant target assemblies, experimentally constrained dynamics, and functional processing and exposure, while retaining negative and discordant findings.

## 1. Introduction

Human immunodeficiency virus type 1 (HIV-1) is an enveloped retrovirus that infects CD4-positive immune cells; without treatment, progressive immune damage can culminate in acquired immunodeficiency syndrome (AIDS), with susceptibility to opportunistic infections and malignancies [1]. The modern epidemic was first recognized through reports of unusual opportunistic illness in 1981, followed by isolation of the virus in 1983 [2,3]. The introduction of potent combination antiretroviral therapy (ART) in 1996 transformed the clinical course of infection, substantially reducing morbidity and mortality [4].

The therapeutic repertoire now spans entry, reverse transcription, integration, protease activity and capsid function. Nevertheless, viral persistence, lifelong treatment, adherence difficulties, adverse effects, drug interactions, resistance and unequal access continue to complicate care [1]. The broader prevention, treatment and cure framework discussed by Landovitz and colleagues emphasizes continuing unmet needs despite the success of antiretroviral therapy [5]. These challenges sustain interest in agents with distinct mechanisms and useful properties for combination regimens.

HIV-1 protease inhibitors provide a particularly relevant medicinal-chemistry precedent. Peptidomimetic active-site inhibitors and subsequent heterocycle-containing designs exploit structural features of substrate recognition. Ghosh and colleagues discuss the development of nonpeptidic inhibitors designed to strengthen interactions with protease backbone atoms, while Funicello and colleagues review the contribution of heterocyclic scaffolds to protease-inhibitor design [6,7]. This established enzyme-directed approach provides the scientific context for examining a complementary strategy that acts on the substrate undergoing maturation.

A small molecule can inhibit a viral enzyme by occupying its catalytic site, but it can also prevent that enzyme from processing a structurally protected substrate. Betulinic acid (BA)-derived HIV-1 maturation inhibitors (MIs) exemplify the latter mechanism. Early mechanistic studies of 3-O-(3′,3′-dimethylsuccinyl) betulinic acid, subsequently known as bevirimat, PA-457, DSB, or MPC-4326, linked antiviral activity to impaired conversion of the capsid–spacer peptide 1 (CA–SP1) intermediate into mature capsid. This disrupted core formation without the broad cleavage inhibition characteristic of a protease active-site inhibitor [8,9]. The distinction is central: the drug-sensitive entity is not simply a peptide sequence but a substrate presented within an assembled and dynamically regulated lattice of the group-specific antigen (Gag) structural polyprotein.

The medicinal-chemistry history began before the target could be visualized at high resolution. Early BA modification studies established potent anti-HIV derivatives, while later biochemical investigations identified the maturation-specific activity of the bevirimat lead [8,10,11]. Successive chemical programs then pursued greater antiviral breadth, lower sensitivity to serum proteins, improved exposure, and more useful dosing characteristics. These efforts generated several related development candidates, including BMS-955176/GSK3532795, GSK3640254, and VH3739937/VH-937 [12,13,14,15]. Their progression supplies a useful test of a common assumption in structure-guided discovery: that a better molecular interaction will necessarily produce a better medicine.

Betulinic acid is a plant-derived pentacyclic triterpene whose semisynthetic derivatives illustrate how a natural-product scaffold can support distinct antiviral mechanisms. Broad reviews of BA chemistry and anti-HIV triterpenoids describe both entry-active and maturation-directed branches [16,17,18,19]. The natural-products medicinal-chemistry account by Lee places bevirimat within a strategy linking a biologically active lead to chemical optimization [20]. For the present question, the relevant unit is therefore a chemically identified derivative with a maturation hypothesis or mechanism, rather than the plant source or the triterpene class alone.

Earlier reviews established the therapeutic rationale for targeting maturation and summarized bevirimat pharmacology, early structure–activity relationships (SAR) and progression to later-generation inhibitors [21,22,23,24,25]. Broader Gag and maturation reviews encompass additional assembly targets, capsid-condensation inhibitors and other mechanisms [26,27,28,29]. Integrative structural biology has also explicitly connected experiment and computation for HIV assemblies [30]. Accordingly, this review does not claim that combining these disciplines is unprecedented. Its contribution is a BA-lineage-focused assessment of whether chemical and computational explanations are supported by matched structural, processing and antiviral observations, extended to the later clinical publications.

Structural biology has substantially changed the interpretation of this chemistry. Cryo-electron tomography and crystallography established the CA–SP1 six-helix bundle as an assembly and maturation switch [31,32]. Microcrystal electron diffraction (MicroED) and solid-state nuclear magnetic resonance (NMR) subsequently provided direct evidence for bevirimat binding within the bundle [33,34]. In parallel, NMR and molecular dynamics (MD) suggested that maturation inhibition involves altering conformational populations and restricting motions needed for processing [35,36]. These developments make it possible to revisit older SAR, but they do not justify assigning every historical analogue a binding pose or a validated mechanism.

This review asks which chemical changes in the BA lineage improve maturation inhibition, how structural and computational evidence explain their effects, and where experimental findings substantiate or limit those explanations. Its focus is deliberately narrower than a general review of BA bioactivity or all HIV capsid-directed agents. BA entry inhibitors, compounds acting at reverse transcriptase or protease, and unrelated triterpenoids are not treated as maturation-inhibitor evidence solely because they inhibit HIV replication. Other scaffolds are considered only where they illuminate a specific mechanistic comparison.

**Figure 1:**
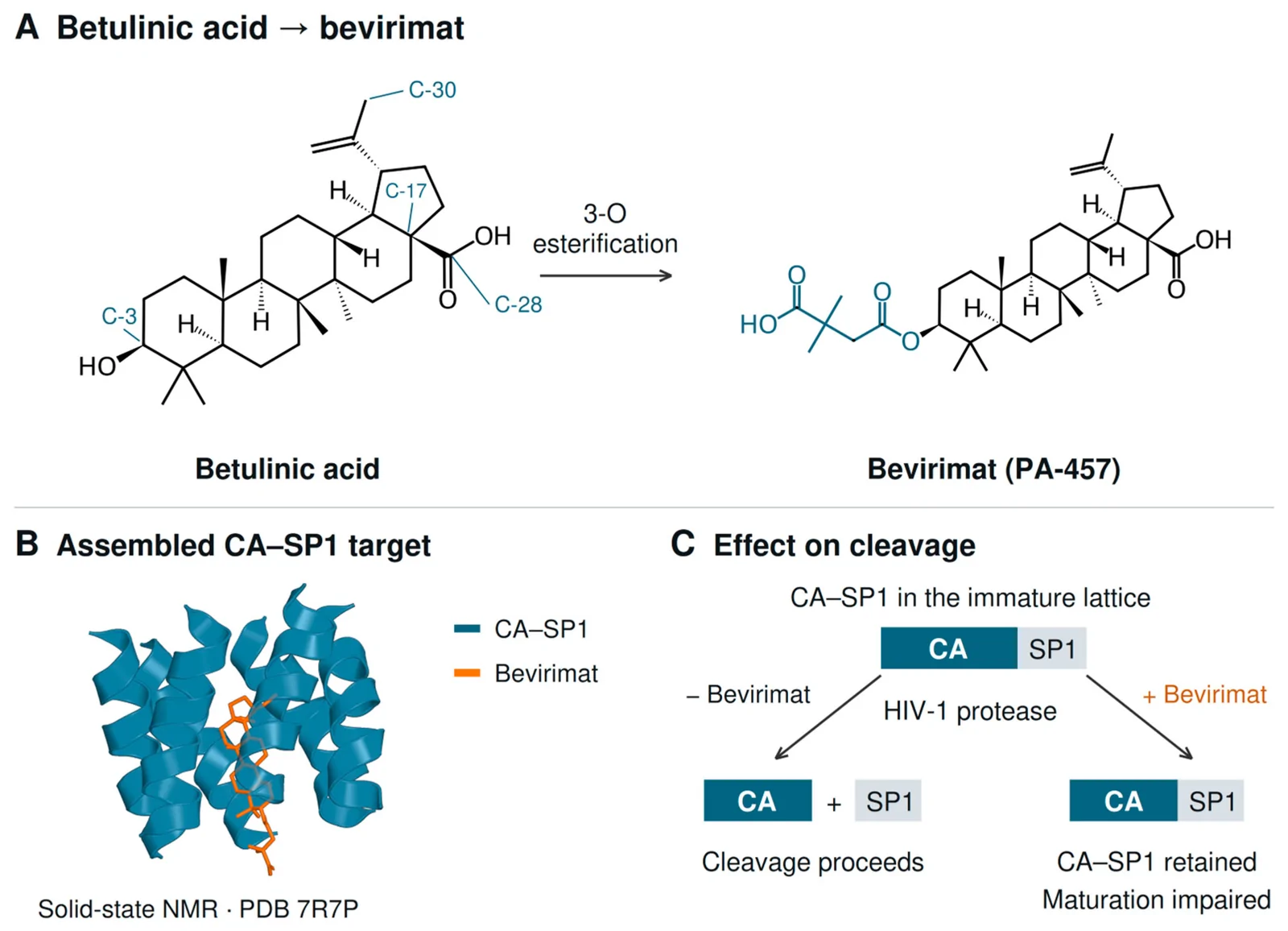
Bevirimat: chemical structure and mechanism of action. Original figure prepared for this review. (**A**) Chemical structures newly drawn from PubChem records for betulinic acid (CID 64971) and bevirimat (CID 457928). Conventional lupane positions C-3, C-17, C-28 and C-30 are annotated on the parent scaffold; the added 3-O ester group is blue. (**B**) New rendering of model 1 of the published solid-state NMR structure, Protein Data Bank (PDB) entry 7R7P [34]. The capsid–spacer peptide 1 (CA–SP1) junction bundle is blue and bevirimat is orange; inositol hexakisphosphate (IP6) and the remaining CA domains are omitted. PDB coordinate data are available under CC0. (**C**) Original schematic illustrating inhibition of CA–SP1 cleavage and maturation [8,9]. No published figure panels were reproduced.

The principal argument is that an effective evidence bridge must connect the same compound, a relevant target assembly, a meaningful dynamic or binding observation, a functional cleavage phenotype, and an antiviral outcome. The literature strongly supports this bridge for the bevirimat mechanism as a class concept. Its extension to individual chemical series, predictions of variant coverage, and clinical performance is less complete. Figure 1 connects the chemical structure of bevirimat with its binding site and inhibition of CA–SP1 processing.

### Literature Search Strategy

To examine how chemical design, target structure and molecular dynamics relate to the experimental and translational evidence for BA-derived HIV-1 maturation inhibitors, literature searches were conducted in Scopus, Web of Science, PubMed, the Cochrane Library and Embase. Primary reports were considered when they addressed chemically defined BA-lineage maturation inhibitors through medicinal chemistry, antiviral activity, target mechanism, susceptibility variation, computation, structural biology, pharmacokinetics or clinical investigation. Studies of CA–SP1 structure and dynamics were also considered when necessary to interpret the inhibitor mechanism. Generic anti-HIV activity or structural similarity alone was insufficient for inclusion in the maturation-focused synthesis. Secondary publications supported background and interpretation, while other scaffolds provided comparative context. The review methodology was registered on the Open Science Framework (DOI: 10.17605/OSF.IO/WNCPM) [37].

## 2. Medicinal Chemistry of Betulinic Acid-Derived Maturation Inhibitors

### 2.1. Early Scaffold Activity and the C-3 Maturation Pharmacophore

The first interpretive task is to separate a chemical scaffold from a mechanism. BA can support derivatives with different antiviral activities, and an anti-HIV readout does not identify the relevant stage of inhibition. The early DSB/bevirimat series is important because the chemically defined lead was subsequently connected to inhibition of CA–SP1 processing, rather than because all BA analogues share that target [8,9,10]. Accordingly, the original potency studies and the later mechanism studies should be read together, with the chronology of evidence preserved.

Modification at C-3 provided a major route to establishing and reshaping the maturation pharmacophore. Studies of monomethylsuccinyl stereoisomers and conformationally restricted derivatives showed that local geometry affects antiviral activity [38,39]. The interpretation becomes stronger where chemical variation is accompanied by a processing assay, as in the selected analogue examined in the 2010 study [39]. It remains weaker where the evidence consists only of replication inhibition. Repetition of an active compound in a later SAR table is useful as a within-study control but does not constitute a second independent discovery.

The C-28 region illustrates why site-based rules require caution. Both maturation-active and entry-active BA derivatives have been reported, including bifunctional compounds designed to influence more than one stage of infection [40]. Dang and colleagues’ 2012 entry-inhibitor series therefore belongs outside the core maturation evidence, even when the compounds retain activity against viruses less susceptible to bevirimat [41]. Conversely, their subsequent maturation-focused work combined antiviral observations with processing experiments and entry discrimination [42]. The mechanism follows the evidence for the individual compound, not the label “C-28 modified.”

### 2.2. C-3 Pharmacophore and C-28 and C-17 Modifications

Replacement of bevirimat’s C-3 dimethylsuccinyl region by a benzoic-acid-containing substituent provided a different geometrical presentation of the acidic pharmacophore. The corresponding studies identified potent compounds with lower serum-associated shifts in activity and established a platform for further modification at the opposite end of the scaffold [43,44]. The medicinal-chemistry achievement was therefore broader than an improved nominal half-maximal effective concentration (EC50): chemical changes altered the balance between antiviral activity and exposure-related properties.

The distinction between C-28 and C-17 nomenclature should remain explicit. C-28 acid/amide transformations and compounds with an amino substituent directly attached in the C-17 region are related through the scaffold but are not interchangeable descriptions of the same bond connectivity. The BMS-955176/GSK3532795 program combined its C-3 substituent with a carefully selected C-17 amine region [12,13]. In a complementary C-28 amine-based series, Chen and colleagues reported compound 20 with a wild-type EC50 of 1.0 ± 0.4 nM, compared with 10 ± 11 nM for bevirimat in that assay. Addition of 15 mg/mL human serum albumin shifted the reported values to 2.0 and 970 ± 710 nM, respectively [45]. These within-study comparisons are more informative than comparing the lowest published values across laboratories.

Alkyl-amine derivatives and subsequent linker-length studies extended the investigation of this region [46,47]. Their results support the importance of side-chain geometry within a defined series; they do not establish a universal rule that a longer linker, a more basic amine, or a more hydrophobic substituent will be advantageous. Changes that improve a replication endpoint can also alter solubility, protein binding, exposure, and tolerability. A meaningful SAR must therefore track these properties alongside the compound-specific mechanistic evidence.

The evolution toward GSK3640254 involved further replacement of the C-3 aromatic element with a substituted cyclohexene carboxylic-acid region [14,48]. Later C-17 amine exploration broadened the chemical options around that lead [49]. VH-937 introduced another modification adjacent to the acidic pharmacophore and combined a strong preclinical antiviral profile with pharmacokinetic properties compatible with investigating less frequent dosing [15,50]. These are documented medicinal-chemistry advances, but retrospective assignment of their entire benefit to a single modeled interaction would exceed the available evidence.

### 2.3. Alternative BA-Lineage Designs and Chemical Developability

Alternative bevirimat-related designs include fluorinated analogues, macrocyclic lupane derivatives, alpha-ketoamides, and compounds incorporating additional polar or privileged fragments [51,52,53,54]. Such work demonstrates that a retained triterpenoid core does not preclude substantial chemical diversification. However, the evidential depth varies: some studies support the mechanism through CA–SP1 processing, whereas others primarily extend phenotypic SAR. The distinction should remain visible in both narrative conclusions and tabulated summaries.

The larger BA analogue series reported in 2021 extends phenotypic chemical coverage and combines synthesis with a quantitative structure–activity relationship (QSAR) analysis [55]. Earlier triterpene work assigned some derivatives a probable late-stage mechanism without direct target localization [56]. These contributions are relevant to the maturation-directed discovery history, but their evidence should not be described as equivalent to ligand localization in assembled CA–SP1. The polar and boron-dipyrromethene (BODIPY)-labeled series is also informative because the reported processing phenotypes differed among compounds [57]. Such within-series differences argue against transferring a single mechanism to every molecule that shares the BA core.

The phosphorus-containing analogues reported by Chrobak and colleagues are a particularly useful bridge case. Compound **14a** inhibited HIV replication in CEM-T4 cells at a half-maximal inhibitory concentration (IC50) of 0.02 ± 0.01 μM, compared with 0.03 ± 0.009 μM for bevirimat; its reported cytotoxicity limit and selectivity were favorable in the same study [58]. A related phosphate study also combined antiviral measurements with docking against the capsid C-terminal domain–SP1 (CTD–SP1) [59]. These articles are relevant to maturation-directed chemistry, but the modeled pose does not establish target engagement or cleavage inhibition for every analogue. The correct conclusion is that the biological results and target hypothesis are compatible and warrant interpretation together, while direct mechanistic confirmation remains incomplete.

EP39 provides a more developed sequence of evidence, progressing from chemistry and processing studies to peptide interaction work and a later solution-NMR analysis of CA–SP1 dynamics [60,61,62]. Even here, the constructs and solvent environments differ across experiments. The apparent continuity of a compound name must not obscure differences in the biological entity being measured.

Chemical developability also matters. Primary process studies addressed access to BMS-955176 and its intermediates, and analytical work corrected assignments of over-alkylation products [63,64,65,66]. These reports support manufacturing feasibility and chemical identity; they do not add independent antiviral efficacy. More recently, isotopically labeled bevirimat derivatives were prepared as NMR probes [67]. This is an enabling advance toward stronger ligand-specific structural evidence, not itself proof that a new compound–target binding structure has already been established. Table 1 summarizes the principal design lessons while retaining these distinctions. Table 1A shows the generic structures, source-specific substituent examples and the full structure of compound **14a**; Table 1B summarizes the broader design evidence.

Enabling chemistry includes process-oriented esterification work directed toward bevirimat manufacture [68]. Its relevance is practical access to a defined inhibitor, rather than a new antiviral result. Likewise, chemical-probe preparation and backbone-resonance assignments are resources for later mechanistic tests [69]. They should be distinguished from completed measurements of inhibitor occupancy or binding thermodynamics. This separation preserves useful synthetic and analytical evidence without inflating the number of independent demonstrations of the biological mechanism.

**Table 1:** Chemical structures and design themes in the BA-derived maturation-inhibitor lineage. (**A**) Generic and representative structures with source-specific analogues. (**B**) Chemical-design evidence and limits of interpretation. Compound numbers are specific to each source; activity comparisons retain their original assay context.

(**A**)			
**Chemical Series**	**General or Representative Structure**	**Representative Analogues/Substituents**	**Source**
C-3 aryl series	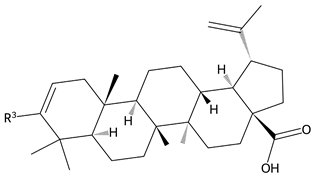	9a: R^3^ = 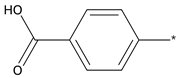 9b: R^3^ = 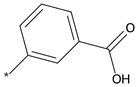	Liu et al. 2016 [43]
C-28 amide series	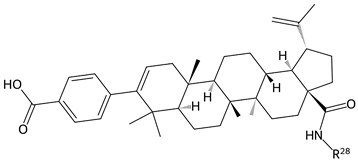	13: R^28^ = CH_2_CH_2_OH 15: R^28^ = CH_3_ 17: R^28^ = 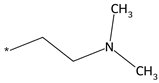	Swidorski et al. 2016 [44]
C-3 replacements/C-17 amine	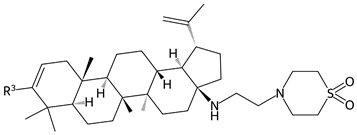	3 (GSK3532795): R^3^ = 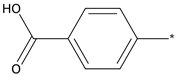 7: R^3^ = 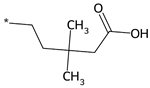	Swidorski et al. 2021 [48]
C-30 phosphonate	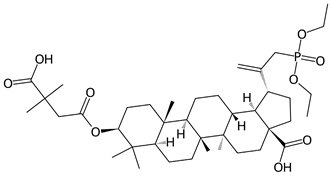	**14a**: full structure shown. Direct C30–P bond. P(=O)(OEt)_2_; C-28 = COOH. C-3: 3-O-dimethylsuccinyl ester.	Chrobak et al. 2019 [58]
(**B**)			
**Design Theme**	**Representative Evidence**	**What the Evidence Supports**	**Important Boundary**
C-3 acidic pharmacophore	Bevirimat; stereochemical and restricted C-3 analogues [10,38,39]	C-3 geometry influences antiviral SAR; selected analogues have CA–SP1 processing evidence.	Early phenotypic SAR does not assign every analogue a maturation mechanism.
C-28 modification	Qian 2012 and Dang 2013 [42,70]	Selected substituents improve within-study potency; entry-discrimination and processing assays refine mechanism.	C-28 substitution can also support entry-active chemistry; classify compound by evidence.
C-28 amine series	Chen 2018, compound 20 [45]	WT EC50 1.0 ± 0.4 nM versus BVM 10 ± 11 nM; albumin-associated shift 2-fold versus 97-fold.	Study-specific cell/albumin conditions; not a clinical exposure comparison.
C-3 benzoic acid/C-17 amine	C-3/C-28/C-17 structures in Table 1A; BMS-955176/GSK3532795 [12,13,43,44,48]	Combined potency, broader tested coverage, and improved pharmacological profile.	C-17 amino substitution is chemically distinct from a C-28 amide; multiple reports overlap.
Cyclohexene-acid region	GSK3640254 and later C-17 analogues [14,48,49]	Further pharmacophore exploration supported broader preclinical activity and exposure.	A specific predicted interaction does not explain the complete clinical profile.
Extended-dosing design	VH-937 [15,50,71]	Medicinal chemistry, preclinical virology, and long human half-life support a dosing hypothesis.	Weekly clinical efficacy is not established by healthy-volunteer PK.
Phosphorus-containing analogues	Compound **14a**, full structure in Table 1A [58]	HIV-1 replication IC50 in CEM-T4 cells: 0.02 ± 0.01 μM versus BVM 0.03 ± 0.009 μM; target-directed modeling.	No direct cleavage/binding confirmation for **14a**; computational method is not unrestricted all-atom MD.
Polar EP39 derivative	Coric 2013; Neyret 2019; Chen 2020 [60,61,62]	Processing, interaction, and NMR observations connect a chemical modification to local dynamics.	Peptide, monomeric protein, and particle experiments are different target contexts.
Alternative lupane designs	Macrocycles and alpha-ketoamides [52,53]	Explicit maturation-focused chemistry expands the structural options within the lineage.	Preserve the evidence level for each series; do not transfer one lead mechanism to all analogues.
Chemical probes/process	BMS-955176 enabling synthesis; labeled BVM probes [64,67]	Chemical identity, access, and ligand-selective NMR tools strengthen future mechanistic work.	Enabling chemistry is not additional independent antiviral or binding validation.

Structures were newly drawn from the cited primary studies. Asterisks mark attachment to the scaffold; R3 and R28 denote substituent variables rather than atom numbers. C-28 amides retain the original carbonyl carbon, whereas the direct C-17 amine series does not retain C-28. BA, betulinic acid; BVM, bevirimat; CA, capsid; SP1, spacer peptide 1; EC50, half-maximal effective concentration; IC50, half-maximal inhibitory concentration; MD, molecular dynamics; NMR, nuclear magnetic resonance; PK, pharmacokinetics; SAR, structure–activity relationship; WT, wild type. CEM-T4 denotes the cell line used in the cited assay.

### 2.4. Comparison Across Development Candidates

Development across the BA lineage has changed several properties at once: the geometry of the acidic pharmacophore, the substituent at the opposite end of the scaffold, antiviral coverage, protein-associated potency shifts, and systemic exposure. Table 2 compares these advances with the limitations that remained. The C-3 and C-17/C-28 changes were often developed together, so a program-level improvement cannot be assigned retrospectively to one substituent without matched-series evidence [12,13,14,15,43,44,45,46,47,48].

Three distinctions are essential to this comparison. Nominal cellular potency is different from activity in protein-containing conditions; activity across a tested variant panel is different from a clinical resistance barrier; and longer exposure is different from a validated dosing regimen. Bevirimat established the mechanism, later candidates broadened the tested antiviral profile, and human studies exposed additional tolerability, resistance and regimen requirements. These represent complementary stages of evidence rather than a single cross-generation potency ranking [50,72,73,74,75,76,77,78].

**Table 2:** Development gains and remaining limitations across representative BA-derived maturation inhibitors. This is a qualitative comparison of programs, not a numerical ranking across different assays.

Compound	Chemical-Design Change	Antiviral and Susceptibility Evidence	Development Boundary
Bevirimat	3-O-dimethylsuccinyl modification of the BA scaffold.	Established CA–SP1 maturation mechanism, but activity against a reference isolate did not predict coverage of naturally variable Gag backgrounds [8,9,79,80,81,82].	Early human antiviral activity supported the target; heterogeneous baseline susceptibility constrained development [74,83,84].
BMS-955176/GSK3532795	C-3 benzoic-acid pharmacophore combined with direct C-17 amine substitution [12,13].	Broader tested antiviral coverage and lower sensitivity to protein-containing assay conditions were important advances over bevirimat [12,13,85].	The phase IIb regimen still raised gastrointestinal tolerability and backbone-resistance concerns; preclinical breadth did not establish an adequate clinical resistance barrier [75].
GSK3640254	Further modification of the C-3 acid-bearing region, together with optimization of the C-17 substituent [14].	Broader preclinical activity and binding-persistence evidence strengthened the development rationale; selected kinetic measurements used labeled surrogates [73].	Resistance signals during early monotherapy and later regimen differentiation remained separate challenges. Phase IIb antiviral activity did not demonstrate superiority [76,77,78].
VH-937	Further BA-lineage modification adjacent to the acidic pharmacophore, with exposure and dosing objectives [15].	The preclinical profile supported further study, but conclusions for a challenging background depended on the assay format [50].	Human pharmacokinetics supported investigation of extended dosing intervals; durable weekly efficacy and regimen-level resistance protection were not established by the phase I study [71].

Compound identifiers are retained as reported. Antiviral breadth, experimental resistance and regimen-level clinical resistance are distinct outcomes.

## 3. Structural and Dynamic Basis of CA–SP1 Inhibition

### 3.1. Structural Evidence for the Six-Helix Bundle Mechanism

Biochemical studies established early that bevirimat activity depends on the structural context of Gag. Drug association with immature particles, photoaffinity mapping, and experiments with assembled substrates connected the compound to the region surrounding the maturation cleavage junction [86,87,88]. Cryo-electron tomography further demonstrated bevirimat-associated persistence of the immature lattice [89]. These observations are more specific than a late-stage antiviral phenotype, because they link drug action to an assembled target and a particular processing event.

The structural framework became clearer when crystallography and cryo-electron tomography resolved the immature CA–SP1 assembly. The junction forms a six-helix bundle in which the cleavage region is not constitutively exposed as a freely accessible peptide [31,32]. Solid-state NMR in immature-like particles independently supported helical structure in this region [90]. A drug that stabilizes the bundle can therefore inhibit processing through conformational restraint rather than through direct competition at the protease catalytic site.

Purdy and colleagues’ MicroED structure provided direct ligand-associated evidence: a bevirimat molecule occupies the central bundle cavity, where the triterpenoid and acidic substituent contribute different classes of interaction [33]. Subsequent atomic-resolution magic-angle-spinning NMR examined bevirimat with and without the assembly cofactor inositol hexakisphosphate (IP6), strengthening the structural basis for interpreting ligand binding and restriction of the junction region [34]. More recent in situ cryo-electron microscopy (cryo-EM) work showed bevirimat and lenacapavir at distinct sites in an immature particle context [91]. This is a justified use of another scaffold: it distinguishes molecular sites and concurrent occupancy rather than enlarging the review into a catalogue of all capsid inhibitors. Structural co-occupancy alone does not demonstrate therapeutic synergy.

### 3.2. Experimental Constructs and Target Assembly States

A CA–SP1 fragment in solution, an assembled tube, a microcrystal, a virus-like particle (VLP), or an immature virion are not equivalent targets. Solution-NMR studies found considerable flexibility in unassembled CA–SP1-containing fragments [92,93]. In the latter study, a detectable bevirimat interaction was not observed under the soluble monomer/dimer conditions examined [93]. This negative result should not be discarded, but neither does it negate binding to the assembled lattice: it tests a different target state under additional solubility constraints.

Likewise, local helicity in a synthetic peptide can depend on concentration, solvent, or an oligomerizing fusion partner [94,95]. These studies explain how the junction may acquire structure, but they cannot reproduce the full geometry, cooperativity, or chemical environment of an authentic immature lattice. The experimental model determines whether an observation concerns local conformational propensity, intersubunit organization, or ligand occupancy in a particle.

This issue remains important even for sophisticated structural preparations. The 2026 cryo-electron tomography (cryo-ET) comparison by Preece and colleagues found differences in lattice organization between Gag-only VLPs and immature HIV-1 particles [96]. Earlier lattice-edge work also showed that finite assemblies include partial hexamers and discontinuities, features absent from an ideal isolated symmetric hexamer [97]. Consequently, a high-resolution structure can accurately describe its specimen while incompletely representing the distribution of states relevant to maturation in a particle. Resolution and biological representativeness are separate strengths.

The historical structural literature also explains why apparently different SP1 descriptions can coexist. Early cryo-EM and cryo-ET studies resolved immature-like assemblies and maturation intermediates [98,99], whereas magic-angle-spinning NMR (MAS NMR) investigations of tubular CA–SP1 assemblies described substantial SP1 mobility and conformational plasticity [100,101]. Structural reorganization accompanying proteolysis was examined in another assembled preparation [102]. These observations concern different specimens and stages. Their interpretation requires preservation of construct, lattice geometry, temperature and processing state, rather than treating every CA–SP1-containing sample as the same six-helix target.

### 3.3. IP6 and Assembly-Dependent Inhibition

IP6 is part of the mechanistic interpretation, not merely an optional additive in a simulation. Primary structural and functional work demonstrated its contribution to immature assembly and mature capsid formation [103]. Further studies linked lattice stability to IP6 packaging and showed that its effects depend on the assembly context [104,105]. These findings explain why “stabilization” is not synonymous with “antiviral action.” Stabilization can support productive assembly at one stage, while excessive or mistimed stabilization can impede later rearrangement and processing.

These observations support a model in which the drug changes the trajectory and timing of an assembly transition. NMR and MD studies of bevirimat point toward restricted junction motions and a shift in the accessible conformational ensemble [34,35,36]. The evidence supports this mechanism at the level of compatible structural and functional observations. It does not imply that one global stability metric will rank all compounds or all target variants. Table 3 contrasts the principal experimental and computational models and the limits of the claims they can support.

### 3.4. Mechanistic Comparisons with Other Scaffolds

PF-46396 provides a useful non-BA comparison because its initial characterization and subsequent clade-specific work linked activity to the same processing junction [111,112]. Its SAR study further showed that association with immature particles and antiviral activity can be dissociated [113]. The later MAS NMR study supplies a direct structural comparison of a different scaffold in an IP6-containing CA–SP1 assembly [114]. These reports strengthen the distinction between binding, stabilization and functional inhibition; they do not establish that BA analogues must share the same pose or sensitivity profile.

Other comparisons define the boundary of the maturation label. The C1 capsid study described impaired maturation without the same Gag-processing phenotype as bevirimat [115], while fullerene derivatives affected late-stage processing without direct protease inhibition [116]. Lenacapavir susceptibility in cleavage-site variants and the concurrent-occupancy structural report address distinct capsid-targeting mechanisms [91,117]. These are comparisons of mechanism and target context, not additions to the BA-derived chemical series.

Beesioside-I-derived compounds provide a particularly relevant natural-product comparison because their study combines synthesis, antiviral evaluation, QSAR, docking and MD [118]. They remain a different scaffold. A similar computational workflow or overlapping processing endpoint is insufficient to transfer a binding mode, dynamic explanation or quantitative SAR between beesioside and BA derivatives.

## 4. Computational Approaches and Experimental Validation

### 4.1. Molecular Docking and Target Hypotheses

Docking is useful for testing whether a chemically plausible pose can be accommodated within a relevant CA–SP1 structure. Its contribution is greatest when the ligand identity, protonation, target assembly, and cofactor state are specified and when the prediction can be compared with an independently measured interaction or functional result. A favorable docking score alone does not demonstrate affinity, target occupancy, inhibition of CA–SP1 cleavage, or antiviral selectivity.

Chrobak’s phosphorus analogue study illustrates both the value and the boundary of this approach [58]. It connects real synthesis and antiviral measurements to a defined target hypothesis, making it more informative than a docking-only screen. However, its flexible-ligand/rigid-target docking calculations and coarse-grained protein-motion analysis should not be described as an unbiased all-atom simulation of a fully solvated inhibitor–bundle complex. Model resolution is consequential: a method that explores ligand poses or protein flexibility cannot automatically establish a physiological binding pathway or a residence time.

The 2024 subtype-modeling report combines homology modeling, docking, MD, and end-point energy calculations [110]. It is directly relevant computational evidence, but agreement between its own methods is not independent experimental validation. Similarly, the 2025 IP6 study couples modeling with biophysical binding assays and therefore adds an orthogonal experimental layer [119]. The bevirimat arm is directly relevant here; DSC, a beesioside-I-derived cycloartane, provides non-BA comparative context. Biophysical enhancement of binding is not, by itself, cellular synergy or a clinical benefit.

### 4.2. Molecular Dynamics and Assembly Models

Static structures show where a ligand can be located; MD asks how the surrounding molecular system behaves over time within the assumptions of the model. Pak and colleagues’ simulations and free-energy analysis associated bevirimat action with preservation of bundle symmetry and restriction of conformational changes [36]. This provides a physically coherent interpretation of the structural observations and processing mechanism. Nevertheless, the simulated distributions and barriers depend on the starting assembly, force-field representation, cofactor treatment, and sampled transitions.

The most persuasive dynamic evidence is cross-method concordance. Wang and colleagues combined NMR, cryo-EM, and MD to show that maturation inhibitors stabilize helical character within a dynamically exchanging CA–SP1 system [35]. The independent experimental component makes the dynamic interpretation stronger than an isolated simulation. However, the tubular assembly used in that investigation represents features of an intermediate state and should not be treated as a complete reconstruction of the native immature particle.

A particularly instructive comparison comes from Gupta, Louis, and Tycko. In spherical VLPs, bevirimat strongly inhibited CA–SP1 cleavage while producing relatively small changes in average NMR chemical shifts; relaxation measurements nevertheless indicated altered junction motions [106]. Large functional effects therefore need not require a large static rearrangement. This strengthens a dynamic interpretation while cautioning against equating a single structural displacement or simulation fluctuation metric with the experimentally measured processing response.

The EP39 literature supplies a complementary example. Its later solution-NMR study reports stabilization of transient helical elements in an engineered monomeric CA–SP1–nucleocapsid (NC) construct [62]. This is evidence for altered local dynamics in that construct, not a direct measurement of binding affinity within an intact six-helix bundle. The broad range of residue-based fitted dissociation constants and reported ligand precipitation further discourage treating those estimates as a single transferable thermodynamic affinity. Earlier peptide measurements also used a helix-favoring solvent environment [60,61]. Across these reports, convergence on dynamic modulation is meaningful, whereas literal equivalence of target states is not.

Coarse-grained studies address another scale. Simulations of lattice growth and IP6-dependent assembly explain how collective organization, curvature, and kinetic trapping may arise [107,108]. Full-length Gag simulations add information about coupling between domains and assembly state [120]. Such models can identify why an isolated local binding site may be insufficient to describe maturation. They do not directly provide atomistic ligand affinity or a validated ranking of medicinal-chemistry candidates. The different computational levels are complementary because they answer different questions.

### 4.3. Quantitative Structure–Activity Relationships

QSAR analysis can summarize associations between chemical descriptors and measured activity, but the biological meaning of the response must be established first. A dataset mixing entry inhibitors, unassigned anti-HIV compounds, and confirmed maturation inhibitors does not automatically support a maturation-specific SAR. Activity units, logarithmic transformations, assay conditions, stereochemistry, and repeated measurements all require attention before a model’s numerical performance can be interpreted.

The 2021 QSAR study by Arief and colleagues reuses a 29-compound BA analogue dataset from an earlier medicinal-chemistry study [45,109]. Its predictions therefore are not new antiviral measurements, and the very small, designated test set limits the precision of external-performance claims. The response transformation used in that analysis, the reciprocal of log EC50, is also sensitive to the concentration unit and has a singularity when the numerical EC50 equals one. A high fit statistic cannot resolve that interpretive problem. The reported subnanomolar value for a proposed analogue should consequently remain a prediction, not enter a table of experimentally measured potency.

Computational susceptibility prediction provides a second, distinct application of modeling. Sequence-based and combined sequence–structure classifiers were developed for bevirimat, and later software incorporated maturation-inhibitor prediction among broader antiretroviral tools [121,122,123]. Such studies evaluate associations in their available datasets. Predictive discrimination does not independently establish a binding mechanism or prospective clinical utility. Phenotyping and genotyping reports belong in the evidence map because they define how susceptibility is measured or inferred, with validation restricted to the endpoints tested [124,125].

### 4.4. Implications for Compound Prioritization

Computational methods are most useful for prioritizing clearly testable alternatives within a chemically and experimentally comparable series. Docking can identify poses compatible with known structural constraints, while molecular dynamics can examine whether a proposed explanation is consistent with the sampled conformational ensemble. Quantitative structure–activity models can help prioritize analogues only within the endpoint and chemical space represented by their data. Agreement between these calculations is not independent validation. A useful prediction should specify the observation that could support or contradict it, such as a measured interaction, a change in structural dynamics, or a processing phenotype. Negative results and disagreements across preparations should inform interpretation rather than be omitted from a compound ranking [33,34,35,36,58,62,106,109,110].

Computational claims are most useful when attached to a clearly delimited proposition: a pose compatible with experimental contacts, a change in local conformational populations, or a within-series association with an experimentally measured endpoint. They become less reliable when several inferential steps are compressed into a single assertion that a compound “binds better and therefore will work better.” Table 4 specifies where the available evidence connects these steps and where it remains incomplete.

## 5. Antiviral Susceptibility and Resistance

Naturally occurring variation was a major challenge for bevirimat because potency against a laboratory reference strain did not guarantee activity across clinically encountered Gag backgrounds. Phenotypic and sequence studies documented heterogeneous susceptibility and established that not all variation around the junction has the same functional consequence [79,80,81,82]. Sequence prevalence is nevertheless not a direct clinical-response rate. The distinction is especially important for reports whose titles imply activity in patients but whose primary data are sequence surveys [126].

Early resistance investigations established that reduced drug susceptibility can coexist with differing effects on processing and viral fitness [127,128,129]. Population surveys and laboratory-selected variants therefore answer related but different questions: one describes the distribution of sequence backgrounds, while the other probes possible routes to altered response. Subtype-focused analogue studies add direct antiviral measurements where sequence prevalence alone cannot establish potency [130]. Resistance evidence is most informative for chemical design when its phenotype is compared in the relevant assay and compound background; it is not a universal ranking of substitution tolerance.

Later-generation compounds improved breadth, but the relevant question is not only whether their EC50 is lower. Lin and colleagues linked antiviral behavior to maximal inhibition, inhibitor binding/dissociation, and substrate cleavage kinetics [72]. Their model explains why incomplete suppression can persist even at high nominal drug concentration and why sustained inhibition over time can matter. This is one of the most substantive bridges in the literature because the model is constrained by several classes of experimental observation rather than by a docking score alone.

The GSK3640254 preclinical characterization further connected potency with improved binding persistence and a broader tested profile [73]. A technical qualification is necessary: radioligand kinetic experiments used labeled surrogate structures, and the corresponding kinetic estimates should not be presented as unqualified measurements of the untouched clinical compound. This does not remove their mechanistic value; it defines what was measured.

The later-generation susceptibility studies also show why a development narrative should avoid declaring resistance “solved” [50,131,132]. In the VH-937 report, conclusions differed between single-cycle and multiple-cycle assay formats for a challenging viral background [50]. Such discrepancies are informative rather than inconvenient: they reveal that assay design can affect the apparent relationship between compound binding, processing, and propagation. No single assay supplies a universal measure of the barrier to treatment failure.

Finally, incomplete processing can have a non-linear effect on particle function. Studies of CA–SP1-containing intermediates demonstrate that a minority of incompletely processed material can interfere with infectivity [133,134]. Consequently, biochemical cleavage inhibition and cellular antiviral inhibition need not have equal numerical magnitudes. A mechanistic synthesis should explain this difference rather than treat it as a failure of one assay or extrapolate a fixed occupancy-to-efficacy conversion across all compounds.

## 6. Pharmacokinetics and Clinical Development

### 6.1. Early Clinical Proof of Concept

The first clinical bevirimat publications established that maturation inhibition could produce a measurable antiviral effect in humans. In a 24-participant single-dose study, the average maximum viral-load reduction exceeded 0.45 log10 copies/mL at the 150- and 250 mg doses, with a mean estimated half-life of approximately 60 h [74]. These observations supported a clinically relevant mechanism, but the study was too small and short to establish durable combination-regimen performance. Phase I pharmacokinetic and tolerability studies provided additional exposure information without resolving the problem of heterogeneous baseline susceptibility [83,84].

Preclinical work supplied complementary evidence before and alongside human development. The bevirimat mouse study connected antiviral effects with a processing readout in that model [135]. QF-036 has separate pharmacological and repeated-dose toxicology publications [136,137]. These reports broaden the development evidence beyond the best-known clinical candidates. However, animal exposure and toxicity observations cannot be substituted for human efficacy or safety, and the tested viral panels do not establish population-wide coverage.

GSK2838232 supplied another proof-of-concept example. In the 33-participant phase IIa study, 10 days of treatment with cobicistat-supported dosing produced a mean maximum decline of approximately 1.70 log10 copies/mL in the 200 mg group [138]. The small cohorts, short duration, pharmacokinetic boosting, and emergence of reduced-susceptibility signals limit extrapolation to a long-term regimen. The accompanying formulation and food-effect studies reinforce that exposure is part of the therapeutic mechanism in practice, even though it is not a property of the binding site itself [139,140].

BMS-955176/GSK3532795 combined broader preclinical activity with a strong short-course clinical signal [85,141]. Its later 206-participant phase IIb trial is more instructive for translation. At week 24, 76–83% of participants receiving GSK3532795 with tenofovir/emtricitabine achieved HIV-1 ribonucleic acid (RNA) below 40 copies/mL, compared with 77% in the efavirenz-based comparator group. Nevertheless, gastrointestinal adverse events occurred more frequently with GSK3532795, and treatment-emergent resistance affecting the nucleoside/nucleotide reverse transcriptase inhibitor (NRTI) backbone was an important concern [75]. An active molecule can therefore fail to provide an acceptable overall regimen, despite favorable potency and a validated target.

### 6.2. GSK3640254 and Combination-Regimen Evidence

The phase IIa GSK3640254 study documented a dose–antiviral response, with a maximum decline of approximately 2.0 log10 copies/mL at 200 mg [76]. The initial longer monotherapy period also produced treatment-emergent resistance signals, after which the study used a shorter exposure period. This supports antiviral proof of concept while underscoring why a short experimental monotherapy response cannot establish a clinically adequate resistance barrier.

The 2025 DOMINO and DYNAMIC publications provide the most informative later regimen evidence identified for this review. DOMINO randomized 161 participants to GSK3640254 at 100, 150, or 200 mg plus two NRTIs, or dolutegravir plus two NRTIs. Week-24 Snapshot suppression below 50 copies/mL occurred in 33/40 (83%), 39/43 (91%), and 32/42 (76%) participants in the respective GSK3640254 groups, compared with 33/36 (92%) in the dolutegravir group [77]. DYNAMIC randomized 85 participants to GSK3640254 plus dolutegravir or dolutegravir/lamivudine. The corresponding suppression results were 21/22 (95%), 17/20 (85%), and 17/22 (77%) for the three GSK3640254 doses, versus 18/21 (86%) with dolutegravir/lamivudine [78].

These results support combination-regimen antiviral activity, but the small phase IIb groups and study-specific decision framework do not justify claiming superiority or definitive non-inferiority. The dose-specific percentages were not monotonic, and Snapshot non-response includes outcomes other than confirmed virological failure. Differences in comparator regimen also preclude treating DOMINO and DYNAMIC as interchangeable replications. Their selected populations, 24-week endpoints, and predominance of men limit generalization to all treatment settings [77,78].

Both papers state that the sponsor stopped the studies because the intended daily oral fixed-dose regimen was not considered sufficiently differentiated from existing two-drug daily oral options [77,78]. This explanation must remain distinct from a claim that the mechanism failed or that the studies established unacceptable toxicity. It also illustrates a translational constraint that is rarely captured by docking or MD: a compound can satisfy molecular and virological objectives while lacking a compelling place in the treatment landscape.

### 6.3. Exposure, Safety and Extended-Dosing Potential

Clinical pharmacology can reveal limitations invisible to target-based analysis. The GSK3640254 mass-balance study involved only five healthy men but supplied direct information on absorption and hepatobiliary elimination [142]. Dedicated interaction studies examined coadministration with antiretroviral partners and metabolic probes [143,144,145,146]. Their findings are specific to the tested conditions; a broadly reassuring interaction profile must not be compressed into “no drug interactions.” For example, etravirine alone reduced GSK3640254 exposure in the reported study [146].

The earlier bevirimat metabolism literature identified glucuronide products, enzyme contributions and species-dependent behavior, while rat disposition and a human regional-absorption study addressed different determinants of systemic exposure [147,148,149,150]. This evidence connects chemical functionality to developability without assuming that an in vitro metabolic pathway determines the complete human concentration profile. It also explains why improvements in a serum-adjusted cellular assay must be considered together with absorption, formulation and elimination.

Similarly, the thorough QT-interval investigation differentiated the 100 mg dose from a supratherapeutic 500 mg dose: the latter exceeded the predefined 10 ms threshold for the maximum placebo-adjusted mean change in the Fridericia-corrected QT interval (QTcF), whereas the 100 mg profile did not [151]. This prevents an unqualified claim of absent cardiac effects. Clinical interpretation must retain dose, population and endpoint context (Table 5).

VH-937 illustrates the appeal and remaining uncertainty of infrequent dosing. Its first-in-human study reported an approximately three-day terminal half-life and substantial food/formulation effects, supporting investigation of extended dosing intervals [71]. Medicinal-chemistry and preclinical reports provide complementary rationale [15,50]. However, a long half-life is not evidence of durable weekly efficacy, acceptable exposure in diverse patients, or a sufficiently forgiving regimen. Official status records checked for context list the subsequent phase II study as terminated and a separate planned phase I study as withdrawn; those registry entries are not treated as peer-reviewed efficacy reports [152,153]. The published pharmacological lessons remain informative regardless of program status.

Long-acting delivery can also be pursued through formulation rather than molecular half-life alone. A 2024 GSK2838232 study examined parenteral delivery platforms and demonstrated tunable sustained release in rats [154]. This is primary formulation evidence, not a clinical demonstration of a long-acting regimen. Separating intrinsic pharmacokinetics from delivery-system performance prevents an attractive dosing concept from being mistaken for established treatment benefit.

## 7. Future Directions and Translational Challenges

### 7.1. Convergence of Chemical, Structural and Functional Evidence

The strongest convergence concerns the class mechanism. Chemically defined bevirimat inhibits a specific late processing event; biochemical and particle-associated experiments connect the drug to assembled Gag; structural methods localize it within the CA–SP1 bundle; and dynamic studies provide a coherent explanation for restricted access to a normally regulated cleavage junction [8,9,33,34,35,87]. Together, these observations support an assembly-dependent, substrate-directed mechanism more convincingly than any one method alone.

A second strong connection is the relationship between binding persistence, processing kinetics, and the completeness of antiviral inhibition. Lin and colleagues’ integrated study is particularly informative because it relates different observables within a connected experimental framework [72]. The later GSK3640254 work extends this logic, with the qualification that labeled surrogates were used for selected kinetic measurements [73]. These studies suggest that the durability and context of target engagement can be as important as a concentration-based potency estimate.

The bridge is less complete for many individual chemical modifications. A compound can show improved activity in a cellular assay while its specific orientation, dynamic effect, and exposure advantage remain incompletely resolved. Conversely, a high-resolution complex can establish a pose without proving that the same interaction explains a clinical dose–response relationship. The review therefore treats agreement as claim-specific: structural localization is well supported for bevirimat, while some analogue-specific explanations remain hypotheses.

These evidence domains address distinct questions and require interpretation within the relevant experimental system.

Attention was paid to compound identity, target construct, assembly state, serum conditions, assay duration, computational resolution, experimental comparators, and repeated data across publications. Multiple reports from the same discovery program were retained when they contributed distinct information, but shared SAR values and trial cohorts were not interpreted as independent replication. For example, a discovery report, a detailed SAR article, and a virological characterization can describe overlapping compounds while answering different questions [12,13,85].

Table 6 summarizes how the principal types of evidence contribute to the interpretation of BA-derived maturation inhibitors. Chemical optimization, target localization, dynamic models, functional processing and clinical observations provide complementary information, with the strength of each connection determined by the measurements and validation available.

References identify the literature supporting each evidence domain. Related publications may describe overlapping compounds, experiments or clinical cohorts and should be interpreted together.

### 7.2. Priorities for Experimental and Computational Validation

Future studies would be most informative if chemical identity, structural preparation, computational model, and functional readout were connected explicitly for the same compound series. Publication of model assumptions and negative or discordant observations would permit readers to distinguish a failed hypothesis from an unsuitable assay or target preparation. Data sharing should retain compound identity and stereochemistry, assay units and conditions, and the origin of reused measurements; otherwise, apparently independent computational validations may reflect the same underlying dataset.

Dynamic explanations should be evaluated against observables with an appropriate physical meaning. A lower structural fluctuation metric is not equivalent to slower cleavage, and an end-point energy estimate is not a measured dissociation rate. Experimental structural and binding observations can constrain a model, while processing and cellular assays test whether the predicted consequence has functional relevance. The recent availability of ligand-specific isotopic probes offers one potential way to strengthen such connections, provided probe behavior is established relative to the parent compound [67].

The field also needs clearer treatment of assembly scale. Evidence from microcrystals, tubes, VLPs, and virions should be reported as complementary rather than silently combined into a single idealized target. Lattice edges, cofactor occupancy, and differences between preparations may explain discrepancies that would otherwise be attributed only to ligand chemistry [96,97]. A limited number of well-connected cross-method comparisons may provide more reliable design insight than a larger set of disconnected docking exercises.

Finally, translational objectives should be expressed in the language of an intended regimen: sustained relevant exposure, tolerability, compatibility with partner drugs, and clinically useful dosing characteristics. DOMINO and DYNAMIC demonstrate that successful target pharmacology does not itself establish differentiation from available treatment options [77,78]. That observation should guide interpretation of the field, without diminishing the value of the underlying mechanistic work.

### 7.3. Limitations of the Evidence and This Review

Several limitations constrain this narrative synthesis. Chemistry studies differ in cell systems, protein/serum conditions, exposure durations and endpoint definitions. Structural experiments examine selected or engineered assembly states, while computational studies vary in resolution and sampling. Much of the clinical evidence derives from relatively small sponsor-supported studies with short follow-up. The source set also reflects the stated scope, publication-type decisions and incomplete retrieval of 13 reports. Formal domain-specific risk-of-bias scoring was not applied; methodological limitations were evaluated in relation to individual claims, assays and models. Missed evidence and selection or interpretation errors therefore remain possible. These constraints prevent a defensible cross-study potency hierarchy or a quantitative estimate of how strongly a structural change predicts clinical benefit.

## 8. Conclusions

BA-derived HIV-1 maturation inhibitors connect natural-product medicinal chemistry to a dynamic viral assembly target. The best-supported mechanism involves engagement of assembled CA–SP1 and restriction of conformational changes associated with its processing. Chemical modification has improved antiviral breadth and pharmacological properties, but those gains do not follow automatically from a docking score or a static binding pose. The most informative studies align chemical identity, target context, structural or dynamic evidence, cleavage behavior, and antiviral activity. Clinical publications add a separate requirement: a mechanistically active compound must also support a tolerable, durable, and differentiated regimen. A critical interpretation of this evidence favors experimentally constrained molecular explanations and transparent uncertainty over a simple catalogue of increasingly potent analogues.

## Figures and Tables

**Table 3:** Target preparations and computational scales answer different mechanistic questions. Examples are selected, not an exhaustive catalogue.

Model or Preparation	Representative Reports	Useful Inference	Limit on Interpretation
Peptides and unassembled fragments	Datta; Deshmukh; EP39 NMR [62,92,93,94]	Local helical propensity, segmental mobility, and ligand-associated perturbations.	Solvent, concentration, and engineered monomerization affect behavior; not an intact six-helix binding site.
Tubular CA–SP1 assemblies	Wang 2017 [35]	NMR/cryo-EM/MD convergence on a dynamic helix–coil equilibrium and inhibitor-associated stabilization.	Intermediate-like tubular geometry is not a complete immature virion.
Microcrystals/assembled CTD–SP1	Wagner; Purdy; Sarkar [32,33,34]	Bundle architecture and direct structural localization of BVM, including cofactor context.	Selected ordered states can underrepresent heterogeneity and finite-lattice effects.
VLP and immature-particle structural/functional studies	Schur; Gupta; Wu; Preece [31,91,96,106]	Assembly-associated structure with progressively richer particle context.	VLPs and virions differ; inhibitor-induced functional changes may exceed visible mean structural changes.
Atomistic MD/free energy	Pak 2021 [36]	Conformational ensembles and modeled coupling of ligand binding to bundle dynamics.	Force field, initial state, sampling, and cofactor representation constrain conclusions.
Coarse-grained assembly	Pak 2017 and 2022 [107,108]	Collective lattice growth, curvature, and kinetic trapping at larger scales.	Does not directly measure atomistic ligand affinity or clinical potency.
Homology models/QSAR	Subtype modeling; BA analogue QSAR [109,110]	Testable target/ligand hypotheses and within-dataset structure–response associations.	Model agreement is not experimental replication; endpoint definition and data leakage require scrutiny.

BA, betulinic acid; BVM, bevirimat; CA, capsid; SP1, spacer peptide 1; CTD, C-terminal domain; cryo-EM, cryo-electron microscopy; MD, molecular dynamics; NMR, nuclear magnetic resonance; QSAR, quantitative structure–activity relationship; VLP, virus-like particle.

**Table 4:** Claim-specific connections between molecular explanations and experimental evidence. The final column identifies the inference that remains open.

Proposed Connection	Supporting Observations	Interpretation	Unresolved Step
Bundle binding → maturation inhibition	Assembled-substrate processing, particle association, MicroED, and MAS NMR [33,34,87,88]	Strong convergence for the bevirimat class mechanism.	Exact occupancy-to-infectivity relationship across compounds and preparations.
Restricted dynamics → impaired processing	Integrated NMR/cryo-EM/MD; VLP evidence [35,36,106]	Conformational restraint is compatible with cleavage inhibition.	Quantitative prediction of cleavage from a chosen simulation metric.
Improved side chain → better activity in serum	Matched compound/BVM measurements in Chen 2018 [45]	Chemical changes improve an integrated cell/serum phenotype.	Relative contribution of binding, uptake, solubility, and exposure.
Predicted polar contacts → phosphorus-analogue activity	Measured replication inhibition plus CTD–SP1 docking [58,59]	Plausible target hypothesis for active compounds.	Direct target engagement and cleavage evidence for individual analogues.
Binding persistence → completeness of inhibition	Binding/cleavage/antiviral model; later-generation characterization [72,73]	Kinetics adds information beyond EC50.	Transfer from labeled surrogates and VLP measurements to clinical exposure.
IP6-associated effects → antiviral benefit	Cofactor structures, assembly work, modeling/biophysical assays [34,103,119]	Cofactor state modifies interpretation of the molecular interaction.	Binding enhancement does not establish cellular or clinical synergy.
Long half-life → less frequent treatment	VH-937 human PK plus preclinical profile [15,50,71]	Supports a pharmacological dosing hypothesis.	Durable efficacy, regimen compatibility, and resistance under real dosing conditions.

BVM, bevirimat; CTD, C-terminal domain; SP1, spacer peptide 1; cryo-EM, cryo-electron microscopy; EC_50_, half-maximal effective concentration; IP6, inositol hexakisphosphate; MAS NMR, magic-angle-spinning nuclear magnetic resonance; MD, molecular dynamics; MicroED, microcrystal electron diffraction; NMR, nuclear magnetic resonance; PK, pharmacokinetics; VLP, virus-like particle.

**Table 5:** Selected peer-reviewed clinical evidence. Different regimens, durations, and viral-load endpoints are presented separately; these values must not be pooled or ranked across trials.

Compound/Report	Design and Exposure	Reported Result	Interpretive Limit
Bevirimat [74]	24 adults; single-dose placebo or 75/150/250 mg; 20-day sampling.	Average maximum HIV-1 RNA decline >0.45 log10 copies/mL at 150 and 250 mg; estimated mean half-life 60.3 h.	Small short-term proof of concept; not durable regimen efficacy.
GSK2838232 [138]	33 adults; 10 days with cobicistat; 200 mg group *n* = 8.	Mean maximum decline 1.70 log10 copies/mL in the 200 mg group.	Boosted, small cohorts; reduced-susceptibility signals require caution.
GSK3532795 [75]	206 treated adults; 60/120/180 mg or efavirenz, each with TDF/FTC; 24 weeks.	HIV-1 RNA < 40 copies/mL: 76–83% with MI versus 77% with efavirenz; more GI events and NRTI resistance concerns with MI.	Antiviral activity did not ensure a favorable complete-regimen profile.
GSK3640254 phase IIa [76]	34 participants; short monotherapy exposure across 10–200 mg.	Maximum HIV-1 RNA changes ranged from −0.4 to −2.0 log10 copies/mL.	Longer initial exposure produced resistance signals; duration changed during study.
DOMINO [77]	161 randomized; MI 100/150/200 mg + two NRTIs versus DTG + two NRTIs; 24 weeks.	Snapshot < 50 copies/mL: 33/40, 39/43, 32/42 versus 33/36.	Small dose groups; partial blinding; not a definitive non-inferiority claim.
DYNAMIC [78]	85 randomized; MI 100/150/200 mg + DTG versus DTG/3TC; 24 weeks.	Snapshot < 50 copies/mL: 21/22, 17/20, 17/22 versus 18/21.	Small, selected population; Snapshot non-response is not identical to confirmed failure.
VH-937 phase I [71]	91 healthy participants; single/multiple dosing and formulation/food assessment.	Approximately 3-day terminal half-life; food and formulation materially affect exposure.	PK supports dosing research, not established weekly treatment efficacy.

HIV-1, human immunodeficiency virus type 1; TDF/FTC, tenofovir disoproxil fumarate/emtricitabine; DTG, dolutegravir; 3TC, lamivudine; GI, gastrointestinal; MI, maturation inhibitor; *n*, number of participants; NRTI, nucleoside/nucleotide reverse transcriptase inhibitor; PK, pharmacokinetics; RNA, ribonucleic acid. Treatment-program status is discussed separately from biological efficacy.

**Table 6:** Evidence types and their roles in interpreting BA-derived HIV-1 maturation inhibitors.

Evidence Domain and Principal Contribution	Interpretive Contribution	References
Chemical diversification and early SAR. Early C-3 pharmacophores, bifunctional derivatives, phosphorus-containing, fluorinated and macrocyclic analogues, EP39 discovery, and related BA-lineage series.	Defines within-series structural relationships; processing or target-engagement evidence establishes mechanistic relevance.	[10,11,38,39,40,42,51,52,53,54,55,56,57,58,59,60,155,156,157,158]
Next-generation C-3/C-17/C-28 design. C-3 benzoic acid replacements, C-28 amides/amines and linker length, C-17 modification, and discovery of BMS-955176, GSK3640254 and VH-937.	Links scaffold optimization to antiviral breadth, serum sensitivity and exposure; individual mechanistic explanations require separate confirmation.	[12,13,14,15,43,44,45,46,47,48,49,70]
Enabling synthesis and chemical probes. Manufacturing routes, oxidation/rearrangement chemistry, side-product control, reagent/intermediate preparation and isotope-labeled probes.	Establishes chemical access, identity and probe availability; these advances support subsequent mechanistic experiments.	[63,64,65,66,67,68,159]
Target engagement, cleavage and functional maturation. Gag association, CA–SP1 processing, lattice stabilization, assembly phenotypes, binding kinetics and dominant-negative effects of processing intermediates.	Connects drug association and processing phenotypes to functional maturation inhibition.	[8,9,61,72,86,87,88,89,133,134,160,161,162,163,164]
Experimental structure and conformational dynamics. Cryo-EM/cryo-ET, crystallography, MicroED and NMR descriptions of CA–SP1 architecture, ligand binding, structural transitions and motion.	Locates the target and ligand and constrains conformational explanations within the experimental assembly.	[31,32,33,34,35,62,69,90,91,92,93,94,95,96,98,99,100,101,102,106,165,166,167]
Computational and cofactor/assembly studies. QSAR, docking, MD and sequence/frequency modeling; experimental IP6 and RNA effects on assembly and maturation-relevant states.	Models generate mechanistic hypotheses; cofactor/assembly experiments define target context. Interpretation depends on resolution, sampling and the measured endpoint.	[36,97,103,104,105,107,108,109,110,119,120,168,169,170,171]
Resistance, polymorphisms and antiviral breadth. Selected resistance substitutions, baseline sequence variation, subtype/species differences, protease backgrounds and susceptibility of variant panels.	Identifies assay- and background-dependent activity and resistance; sequence prevalence requires phenotypic interpretation.	[79,80,81,82,126,127,128,129,130,131,132,172,173,174,175,176,177,178,179,180,181,182,183,184]
Assays and susceptibility-prediction methods. Quantitative cleavage analysis, recombinant phenotyping, deep sequencing, sequence/structure classifiers and computational resistance tools.	Defines how susceptibility and cleavage are measured or predicted, with validation specific to the tested endpoints.	[121,122,123,124,125,185]
Preclinical virology, exposure and developability. Candidate virology profiles, animal activity, metabolism, glucuronidation, toxicity and formulation/delivery approaches.	Evaluates animal activity and developability; translation requires separate human pharmacological and clinical evidence.	[50,73,85,135,136,137,147,148,149,154]
Human pharmacokinetics, safety and interactions. Dose exposure, first-in-human evaluation, absorption site/food/formulation effects, metabolism, QT assessment and drug–drug interactions.	Defines dose exposure, tolerability and interactions under the conditions and populations tested.	[71,83,84,139,140,142,143,144,145,146,150,151,186,187,188,189]
Clinical antiviral proof of concept and regimens. Bevirimat and later BA-lineage candidates in people living with HIV, including monotherapy proof of concept and randomized combination-regimen studies.	Tests antiviral responses and regimen performance in humans; study design, duration and comparator constrain interpretation.	[74,75,76,77,78,138,141]

BA, betulinic acid; CA, capsid; SP1, spacer peptide 1; cryo-EM, cryo-electron microscopy; cryo-ET, cryo-electron tomography; Gag, group-specific antigen polyprotein; HIV, human immunodeficiency virus; HIV-1, human immunodeficiency virus type 1; IP6, inositol hexakisphosphate; MD, molecular dynamics; MicroED, microcrystal electron diffraction; NMR, nuclear magnetic resonance; QSAR, quantitative structure–activity relationship; RNA, ribonucleic acid; SAR, structure–activity relationship. QT denotes the electrocardiographic interval used to assess ventricular depolarization and repolarization.

## Data Availability

No new data were created or analyzed in this study. Data sharing is not applicable to this article.

## Abbreviations

The following abbreviations are used in this manuscript:

BA	Betulinic acid
BVM	Bevirimat
CA	Capsid
CTD	C-terminal domain
DTG	Dolutegravir
EC50	Half-maximal effective concentration
HIV-1	Human immunodeficiency virus type 1
IC50	Half-maximal inhibitory concentration
IP6	Inositol hexakisphosphate
MD	Molecular dynamics
MI	Maturation inhibitor
MicroED	Microcrystal electron diffraction
NMR	Nuclear magnetic resonance
NRTI	Nucleoside/nucleotide reverse-transcriptase inhibitor
PK	Pharmacokinetics
QSAR	Quantitative structure–activity relationship
SAR	Structure–activity relationship
SP1	Spacer peptide 1
VLP	Virus-like particle
AIDS	Acquired immunodeficiency syndrome
ART	Antiretroviral therapy
BODIPY	Boron-dipyrromethene
cryo-EM	Cryo-electron microscopy
cryo-ET	Cryo-electron tomography
FTC	Emtricitabine
Gag	Group-specific antigen polyprotein
GI	Gastrointestinal
MAS NMR	Magic-angle-spinning nuclear magnetic resonance
NC	Nucleocapsid
PDB	Protein Data Bank
QT	Electrocardiographic QT interval
QTcF	QT interval corrected for heart rate using Fridericia’s formula
RNA	Ribonucleic acid
TDF	Tenofovir disoproxil fumarate
WT	Wild type
3TC	Lamivudine
